# Two unrelated putative membrane-bound progestin receptors, progesterone membrane receptor component 1 (PGMRC1) and membrane progestin receptor (mPR) beta, are expressed in the rainbow trout oocyte and exhibit similar ovarian expression patterns

**DOI:** 10.1186/1477-7827-4-6

**Published:** 2006-02-03

**Authors:** Brigitte Mourot, Thaovi Nguyen, Alexis Fostier, Julien Bobe

**Affiliations:** 1 Institut National de la Recherche Agronomique, INRA-SCRIBE, IFR 140, Campus de Beaulieu, 35000 Rennes, France

## Abstract

**Background:**

In lower vertebrates, steroid-induced oocyte maturation is considered to involve membrane-bound progestin receptors. Two totally distinct classes of putative membrane-bound progestin receptors have been reported in vertebrates. A first class of receptors, now termed progesterone membrane receptor component (PGMRC; subtypes 1 and 2) has been studied since 1996 but never studied in a fish species nor in the oocyte of any animal species. A second class of receptors, termed membrane progestin receptors (mPR; subtypes alpha, beta and gamma), was recently described in vertebrates and implicated in the progestin-initiated induction of oocyte maturation in fish.

**Methods:**

In the present study, we report the characterization of the full coding sequence of rainbow trout PGMRC1 and mPR beta cDNAs, their tissue distribution, their ovarian expression profiles during oogenesis, their hormonal regulation in the full grown ovary and the in situ localization of PGMRC1 mRNA in the ovary.

**Results:**

Our results clearly show, for the first time in any animal species, that rainbow trout PGMRC1 mRNA is present in the oocyte and has a strong expression in ovarian tissue. In addition, we show that both mPR beta and PGMRC1, two members of distinct membrane-bound progestin receptor classes, exhibit highly similar ovarian expression profiles during the reproductive cycle with maximum levels during vitellogenesis and a down-expression during late vitellogenesis. In addition, the mRNA abundance of both genes is not increased after in vitro hormonal stimulation of full grown follicles by maturation inducing hormones.

**Conclusion:**

Together, our findings suggest that PGMRC1 is a new possible participant in the progestin-induced oocyte maturation in fish. However, its participation in the process of oocyte maturation, which remains to be confirmed, would occur at post-transcriptional levels.

## Background

Steroids may exert their action by two distinct ways 1- a direct control of gene transcription involving classic nuclear receptors [1]; 2- a rapid non-nuclear action, independent of direct regulation of gene transcription [2,3]. This second type of steroid action has been proposed to be involved in steroid-induced oocyte maturation in lower vertebrates. Thus, in amphibians, it has been assumed for a long time, that the actinomycin D-insensitive progesterone-induced oocyte maturation [4] was mediated by a membrane steroid receptor rather than a nuclear receptor. The recent cloning and sequencing of a gene coding for a membranous progesterone receptor [5] in African clawed frog (*Xenopus laevis*) brought evidence for the existence of both intracellular and membrane progestin receptors [6].

In fishes, 2 distinct progestins, 17,20β-dihydroxy-4pregnen-3one (17,20βP) and 17,20β,21-trihydroxy-4pregnen-3one (17,20β,21P), have been identified as the natural Maturation-inducing steroids (MIS), depending on the species [7-9]. High affinity binding for 17,20βP, the MIS in salmonids, has been found in ovarian plasma membranes of rainbow trout (*Oncorhynchus mykiss*) [10], brook trout (*Salvelinus fontinalis*) and Artic char (*Salvelinus alpinus*) [11,12]. This specific 17,20βP binding was high in non-matured post-vitellogenic rainbow trout and medaka (*Orizias latipes*) oocytes, but low in matured oocytes [13]. On the other hand, both nuclear and membrane progestin receptors were fully characterized in the spotted seatrout (*Cynoscion nebulosus*), the membrane receptor being more specific for 17,20β,21P, which is the MIS in this species, than the nuclear receptor which exhibited a higher affinity for 17,20βP [14,15]. Furthermore, membrane receptor capacity of the spotted seatrout ovary has been shown to be correlated with oocyte responsiveness to 17,20β,21P for maturation, assessed by the occurrence of germinal vesicles breakdown (GVBD) [16]. In addition, the cDNA encoding for a putative membrane progestin receptor (mPR) was recently cloned in spotted seatrout [5]. It belongs to a novel family of putative membrane receptors genes present in various vertebrates and displaying three subtypes (α, β and γ) [17]. To date, the 3 forms have been identified in human, mouse, catfish (*Ictalurus punctatus*) and fugu (*Takifugu rubripes*), while 2 forms, α and β, have been reported in pig and zebrafish (*Danio rerio*) and only 1 form in African clawed frog (β), goldfish (*Carassius auratus*) (α) and spotted seatrout (α) [5,18,19]. The recombinant protein produced in an *E. coli *expression system using the spotted seatrout mPRα cDNA sequence exhibited a specific and saturable progesterone binding. Steroid competition studies showed that binding was highly specific for progesterone and 17-hydroxyprogesterone. However, no displacement of tritiated progesterone was observed with 17,20β,21P, the natural spotted seatrout MIS, which has been hypothesized to be due to a lack of adequate post-translational modifications in the *E. coli *expression system [5]. Microinjection of mPRα antisense nucleotide in oocytes resulted in an almost complete inhibition of 17,20βP-induced oocyte maturation in zebrafish [5] while a partial inhibition was observed in goldfish [19]. Similar experiments conducted in zebrafish oocytes using the mPRβ subtype resulted in an almost complete inhibition of oocyte maturation suggesting that mPRβ could also participate in the control of oocyte maturation in zebrafish [20]. Indeed, within the same experiment, the maximum inhibition was observed using the mPRβ subtype [20]. Finally, mRNA tissue distribution of the 3 subtypes have been studied in catfish and mPRβ showed a predominant expression in the ovary [18]. Apart from oocyte maturation, mPRα protein has been detected by Western blotting in Atlantic croaker (*Micropogonias undulatus*) sperm, a species in which a 17,20β,21P action on spermatozoa motility was suggested [21].

Some years ago, a totally distinct progesterone binding protein was isolated from porcine liver microsomal membranes and partially sequenced [22]. This protein, originally termed membrane progesterone receptor, is now termed progesterone membrane receptor component 1 (PGMRC1) [23]. Porcine PGMRC1 cDNA was subsequently cloned [24] as well as the human [25] and rat [26] orthologs. In addition, a related sequence, PGMRC2, belonging to the same family was also reported in humans [25,27]. Human PGMRC1 mRNA has been detected in a wide variety of tissues (Table 1) and has a predominant expression in liver and kidney [25]. In addition, PGMRC1 and PGMRC2 mRNAs have been found in human spermatozoa [27]. PGMRC1 and PGMRC2 cDNAs were also generated from several species subjected to massive cDNA sequencing programs, including mouse, chicken, African clawed frog and zebrafish, and the corresponding sequences deposited in GenBank (Table 1). Antibodies raised against porcine PGMRC1 can mitigate the progesterone induced acrosome reaction [28,29]. PGMRC1 protein was also detected in human spermatozoa [27] and an antibody raised against PGMRC1 could suppress the rapid progesterone-initiated Ca^2+ ^flux observed in sperm. Together, this suggests that PGMRC1, if present in the oocyte, is a candidate for mediating progestin-induced oocyte maturation in fish. However, besides sequences originating from genome sequencing programs, PGMRC1 was never characterized in any fish species. Furthermore, to our knowledge, PGMRC1 protein or mRNA expression was never reported in the oocyte of any vertebrate species (Table 1).

**Table 1:** Sequence accession number, cDNA source or expression data and sequence identity with rainbow trout (rt) PGMRC1 of vertebrate PGMRC1 and PGMRC2 proteins. The tissues exhibiting a predominant expression are underlined.

**Protein**	**GenBank #**	**Species**	**Tissue expression data or cDNA source**	**% sequence identity with rtPGMRC1 (AAL49963)**
PGMRC1	NP_006658	human	heart, brain, placenta, lung, liver, skin, kidney, pancreas	72 %
	NP_999076	porcine	brain, liver, lung, kidney, spermatozoa	71%
	O55022	mouse	liver	71%
	NP_068534	rat	brain, liver	68%
	CAG31527	chicken	2-week embryo	67%
	AAH76926	*Xenopus tropicalis*	whole body (male)	74%
	AAH72727	*Xenopus laevis*	embryo	75%
	CAF97306	*Tetraodon nigroviridis*	-	77%
	AAH85558	zebrafish	liver	89%

PGMRC2	AAH92478	human	heart, brain, placenta, lung, liver, skin, kidney, pancreas	60%
	AAH44759	mouse	mammary tumor	59%
	NP_001008375	rat	-	59%
	XP_420466	chicken	-	59%
	AAH64268	*Xenopus tropicalis*	embryo (tailbud)	62%
	AAH81155	*Xenopus laevis*	brain	60%
	CAG03786	*Tetraodon nigroviridis*	-	63%
	AAH53415	zebrafish	whole body	60%

Therefore, the present study aimed at 1- characterizing the PGMRC1 cDNA sequence in rainbow trout, 2- studying the rainbow trout PGMRC1 mRNA tissue distribution with special interest for any oocyte mRNA expression and, 3- if present in the oocyte, characterizing PGMRC1 mRNA expression profiles during oogenesis. In addition, in order to evaluate PGMRC1 eligibility as a possible participant in the progestin-induced oocyte maturation, we aimed at performing a similar analysis for the so far unknown rainbow trout mPRβ subtype that has recently been implicated in the control of meiosis resumption in zebrafish, a species exhibiting the same maturation-inducing steroid (17,20βP) than rainbow trout. Among mPR subtypes, mPRβ was chosen because it has a predominant expression in catfish ovary [18] and exhibits the maximum oocyte maturation inhibition in antisense oligonucleotide studies [20]. In addition, mPRβ was the only mPR subtype that could be identified in African clawed frog [5].

## Methods

### Animals and tissue collection

Investigations were conducted according to the guiding principles for the use and care of laboratory animals and in compliance with French and European regulations on animal welfare. Rainbow trout were obtained from an experimental fish farm (PEIMA, Sizun, France) and held during spawning season in a recirculated water system, at 12°C, under natural photoperiod (SCRIBE, Rennes, France) until tissue collection. Ovaries were sampled from distinct females exhibiting the following ovarian developmental stages: ovarian differentiation (1000 degree. days after fertilization, N = 5), pre-vitellogenesis (N = 6), early vitellogenesis (N = 6), mid-vitellogenesis (N = 6), late vitellogenesis (N = 6), post-vitellogenesis (N = 6) and final oocyte maturation (N = 5). Late vitellogenic samples were obtained 3–4 weeks before spawning period. Post-vitellogenic samples were obtained during spawning period but before any morphological evidence of oocyte maturation (cytoplasmic clearing or GVBD) [30]. To collect ovarian tissue, trout were deeply anaesthetized in 2-phenoxyethanol, killed by a blow on the head and bled by gill arch section. The abdominal cavity was opened and the ovaries were removed, dissected and aliquots were snap frozen in liquid nitrogen and stored at -80°C until RNA extraction. For the tissue distribution study, different tissues were sampled from 3 post-vitellogenic females. Unfertilized eggs were sampled from 3 freshly ovulated females and testis samples were also obtained from 3 different males at stage II [31] of spermatogenesis.

### cDNA cloning and sequence analysis

PGMRC1 and mPRβ rainbow trout cDNA sequences were identified using a reciprocal blast strategy. A tblastn comparison was performed against all rainbow trout expressed sequence tags (ESTs) available in dbEST [32] using the target amino acid sequence as a query. Comparison was performed using catfish mPRβ as well as porcine PGMRC1 amino acid sequences. The identity of selected cDNAs was subsequently checked using a blastx [33] comparison against the nr database. Corresponding clones were subsequently obtained from INRA-Agenae program resource center (Jouy-en-Josas, France) [34]. Both mPRβ and PGMRC1 clones were fully sequenced and missing 5' sequence of PGMRC1 cDNA was obtained by PCR amplification from a rainbow trout testis cDNA library [35]. The corresponding 650 bp PCR product was cloned in PGEM-T easy vector (Promega, Madison, WI) and sequenced. Subsequently, a PCR amplification was performed in order to confirm the assembled sequence of PGMRC1 cDNA.

Predictions of potential N-glycosylation and phosphorylation sites as well as putative transmembrane domains of PGMRC1 and mPRβ deduced amino acid sequences were performed using the prediction server of the Center for Biological Sequence Analysis . Hydrophobicity analysis of PGMRC1 and mPRβ deduced protein sequences were performed according to Kyte and Doolittle [36] on the Expasy web site .

### RNA extraction and reverse transcription

RNA extraction and reverse transcription were performed as previously described with minor modifications [37]. Briefly, ovarian tissue was homogenized in Trizol reagent (Invitrogen, Cergy Pontoise, France) at a ratio of 100 mg per ml of reagent. Total RNA was extracted using the Trizol procedure and resuspended in water. In order to ensure optimal RNA quality, RNA samples with high yolk contamination were further purified using a Nucleospin RNA II kit according to manufacturer's instructions (Macherey Nalgel, Düren, Germany). Three micrograms of total RNA were reverse transcribed using 200 units of Moloney murine Leukemia virus (MMLV) reverse transcriptase (Promega) and 0.5 μg random hexamers per μg of RNA (Promega) according to manufacturer's instruction. RNA and dNTPs were denatured for 6 min at 70°C, then chilled on ice for 5 min before the reverse transcription master mix was added. Reverse transcription was performed at 37°C for 1 hour and 15 min followed by a 15 min incubation step at 70°C. Control reactions were run without MMLV reverse transcriptase and used as negative PCR controls.

### Real-time PCR

Real-time PCR was performed using an I-Cycler IQ (Biorad, Hercules, CA) as previously described [37]. Reverse transcription products were diluted to 1/50 and 5 μl were used for each real-time PCR reaction. Triplicates were run for PCR reaction. Real-time PCR was performed using a real-time PCR kit provided with a SYBR Green fluorophore (Eurogentec, Belgium) according to the manufacturer's instructions and using 600 nM of each primer. PCR primers were designed in order to generate a short PCR product: PGMRC1 (forward CCCAACCAAGCAGAGAGAAA; reverse AGAGGCCAAGCAGACTCAAA), mPRβ (forward CCCAGCAACAGGTGTGTTC; reverse AAGGAGGGAGGAAAGAGGT) and 18S (forward CGGAGGTTCGAAGACGATCA; reverse TCGCTAGTTGGCATCGTTTAT). After a 2 min incubation step at 50°C and a 10 min incubation step at 95°C, the amplification was performed using the following cycle: 95°C, 20 sec; 62°C, 1 min, 40 times. For each target gene, the relative abundance of cDNA within sample set was calculated from a serially diluted ovarian cDNA pool using the I-Cycler IQ software. Subsequently, real-time PCR data were normalized using 18S RNA abundance. After amplification, a fusion curve was generated in order to ensure that a single PCR product had been generated [38]. The fusion curve was obtained using the following protocol: 10 sec holding followed by a 0.5°C increase, repeated 80 times and starting at 55°C.

### In vitro hormonal regulation

Full-grown ovarian follicles were isolated and incubated in vitro as previously described [37]. Briefly, 25 follicles were incubated at 12°C in 3 ml of mineral medium (IM8/300) (133 mM NaCl, 3.09 mM KCl, 0.28 mM MgSO_4_, 0.98 mM MgCl_2_, 3.40 mM CaCl_2_, 5.55 mM Glucose, 20 mM HEPES, pH 8.0, 300 mOsm) in 6-well culture plates. Follicles were incubated in presence of partially purified gonadotropins (23 or 188 ng proteins/ml), 17,20βP (40 ng/ml) and estradiol (E2, 1 μg/ml). Partially purified gonadotropins were obtained as previously described [39] from an affinity chromatography on a concanavalin A sepharose of a pool of salmon pituitaries sampled during spawning season, a period when luteinizing hormone (LH) is predominantly synthesized. This LH-rich salmon gonadotropin fraction was previously used and the concentration of 188 ng/ml was found to be efficient in inducing 100% in vitro oocyte maturation in most females assayed [37,40]. Similarly, a concentration of 40 ng/ml of 17,20βP was previously used to successfully induce in vitro oocyte maturation in rainbow trout [41]. In addition, a dose of 1 μg/ml of estradiol was previously found to significantly inhibit oocyte maturation in rainbow trout [42]. After a 20-hour incubation, follicles were removed and processed for RNA extraction. Follicles originating from each well were processed separately for RNA extraction. For each treatment, 3 wells were used for RNA extraction from intact follicles. Before in vitro incubation, intact and deyolked follicles were sampled. Follicles were deyolked as previously described by firmly pressing the tissue between 2 mesh screens under constant ice-cold mineral medium aspersion [43]. Intact and deyolked follicle samples were frozen in liquid nitrogen and stored at -80°C until RNA extraction. For each hormonal treatment, 2 additional groups of 25 follicles were incubated for 60 h to monitor GVBD occurrence. The experiment was performed 3 times using follicles originating from 3 different ovaries.

### In situ hybridization

For in situ hybridization, mid-vitellogenic ovarian samples originating from 3 different females were fixed overnight at 4°C in Dietrick's fixative (10% Formaldehyde, 28. 5% ethanol, 2% glacial acetic acid), then rinsed in tap water for 1 hour and held in 50% ethanol. Dehydration (increasing ethanol: 15 min in 50% ethanol, twice 15 min in 70% ethanol, 15 min in 80% ethanol, 30 min in 96% ethanol, and 30 min in 96% ethanol/butanol vol/vol), clearing (butanol once for 30 min, and twice for 3 h each), and paraffin infiltration (once for 1 h and twice for 2 h, at 60°C) were performed in a Citadel 1000 tissue processor (Shandon, Pittsburgh, PA). Dehydrated tissues were embedded in plastic molds in paraffin using an HistoEmbedder (TBS88, Medite, Germany). Tissue sections (6 μm) were cut and placed on poly-lysine treated microscope slides. PGMRC1 Sense (S) and Antisense (AS) riboprobes were generated using a PCR amplification of PGMRC1 clone (1430 bp) in PT7T3 vector, followed by a PCR product purification using a Microcon PCR filter kit (UFC7PCR50, Millipore). S and AS probes were generated using Riboprobe in vitro Transcription Systems (Promega) according to manufacturer's instruction, with 0.35 mM digoxigenin-UTP (Roche Diagnostics Corp, Indianapolis, IN). Hybridization was then performed as previously described with minor modifications [44]. Sections were deparaffined in two washes of Toluene for 10 min each and hydrated in decreasing concentrations of ethanol (100, 95, 85, 70, and 50 %) for 2 min each. They were washed twice 5 min in TBS-Tween (TBS-T, 150 mM NaCl, 50 mM Tris-HCl pH 7. 4, 0. 05 % Tween 20), and fixed 20 min in 4% paraformaldehyde in 0. 01 M PBS. Sections were washed again three times for 2 min in TBS-T, and incubated 10 min at 37°C in TBS/2 mM CaCl_2 _and 1 μg/ml proteinase K. The reaction was stopped in 50 mM Glycine, 50 mM tris-HCl pH 7. 4 for 5 min, and sections were washed 2 min three times in TBS-T. Sections were fixed again 5 min in 4% paraformaldehyde in 0.01 M PBS, and washed 2 min three times in TBS-T. Sections were acetylated 10 min in 0.25% acetic anhydride acid in 0.1 M triethanolamine pH 8, and washed twice in TBS-T and once in TBS. Slides were prehybridized in a humid chamber for 30 min at 50°C in an hybridization buffer (50% formamide, 10% dextran sulfate, 2× SSC, 1× Denhardt's reagent, 0.25 mg/ml yeast transfer RNA). Riboprobes were diluted (8 ng/μL) in hybridization buffer and denaturated for 4 min at 95°C. Sections were then incubated with riboprobes in a humid chamber overnight at 50°C. Sections were washed at room temperature 10 min three times in 2× SSC, incubated for 30 min in 10 μg/mL RNAse A in 2× SSC. They were then washed 15 min at 55°C in 2× SSC, 15 min at 55°C in 0. 1× SSC twice and 5 min in TBS at room temperature. Non-specific sites were blocked 30 min in blocking solution (TBS containing 1% sheep serum), and sections were incubated overnight at 4°C in 1:1000 diluted alkaline phosphatase conjugated digoxigenin in blocking solution. Sections were washed 2 min three times in TBS. After adding NBT (nitroblue tetrazolium chloride) and BCIP (5-bromo-4-chloro-3 indolylphosphate p-toluidine salt), slides were checked regularly and color development was stopped after 4 hours 30 min for both Sense and AntiSense probes. Sections were mounted with Faramount Aqueous Mounting medium (DakoCytomation).

### Statistical analysis

Statistical analyses were performed using Statistica 7.0 software (Statsoft, Tulsa, OK). Differences between ovarian developments stages, comparative abundance between intact and deyolked ovarian tissue and hormonal regulation data were analyzed using non parametric U tests.

## Results

### cDNA sequence

**Figure 1:**
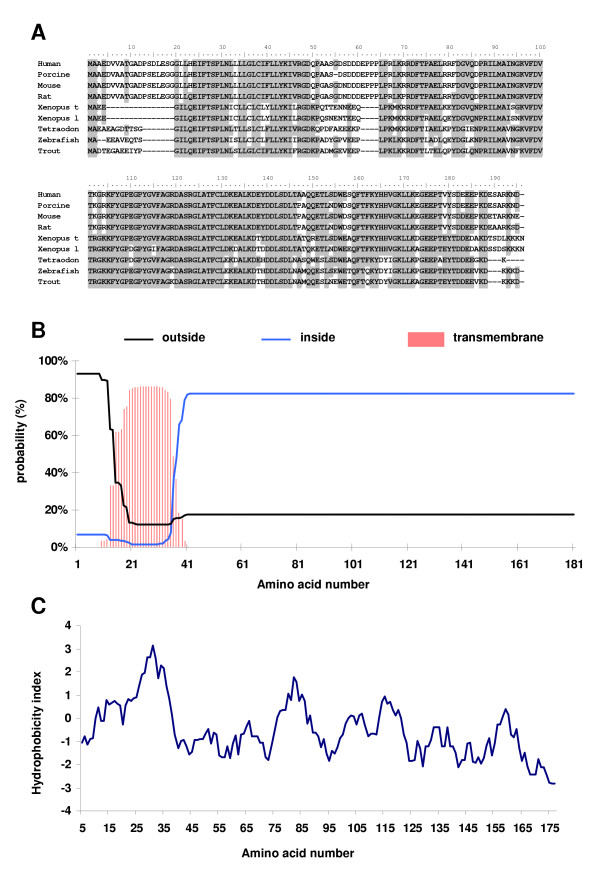
(**A**) Alignment of the deduced amino acid sequence of rainbow trout PGMRC1 with other vertebrates PGMRC1 and PGMRC2 proteins. Most conserved residues are shaded. Corresponding GenBank accession numbers are shown in Table 1. (**B**) Outside, inside and transmembrane probability of rainbow trout PGMRC1 deduced protein. (**C**) Hydrophobicity analysis of rainbow trout PGMRC1 deduced protein sequence according to Kyte and Doolittle [36].

Rainbow trout PGMRC1 cDNA (GenBank AY069921) encoded for a protein exhibiting strong sequence identity with other vertebrates PGMRC1 (72–89%, Table 1, Figure 1A). In addition, rainbow trout PGMRC1 had significant, but lower, sequence identity with other vertebrates PGMRC2 (59–63%, Table 1). The deduced PGMRC1 protein (GenBank AAL49963) was 181 amino acid long. It contained a putative transmembrane domain between aa 13 and 35 (Figure 1B) and a potential N-glycosylation site at position 24. A 26 residue hydrophobic region was found near the N-terminus, between amino acids 13 and 39 (Figure 1C). In addition, a blast over the conserved domain database (CD-search) [45] clearly showed that rainbow trout PGMRC1 contained a cytochrome b5-like/steroid binding domain (aa 62 to 159) previously described in other vertebrates PGMRC1 and PGMRC2 proteins [46].

**Figure 2:**
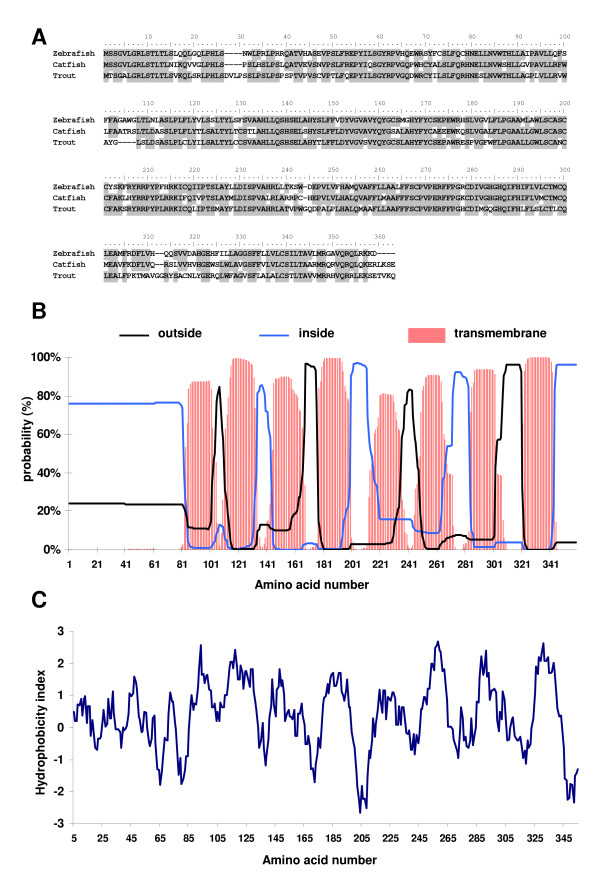
Alignment of rainbow trout (ABA39295), zebrafish (NP_899187) and catfish (AAS45555) mPRβ deduced amino acid sequences. Most conserved residues are shaded. (**B**) Outside, inside and transmembrane probability of rainbow trout mPRβ deduced protein. (**C**) Hydrophobicity analysis of rainbow trout mPRβ deduced protein sequence according to Kyte and Doolittle [36].

Rainbow trout mPRβ cDNA (GenBank DQ191163) encoded for a 359 aa protein (GenBank ABA39295) exhibiting 66% sequence identity with zebrafish mPRβ and 67% sequence identity with catfish mPRβ (Figure 2A). The rainbow trout mPRβ amino acid sequence exhibited 8 putative transmembrane domains at positions 82–101, 111–133, 145–167, 177–199, 212–234, 249–271, 284–301 and 321–343 (Figure 2B). Each putative transmembrane domain corresponded to an hydrophobic region (Figure 2C). In addition, a potential N-glycosylation site was observed at position 80.

### Tissue expression

**Figure 3:**
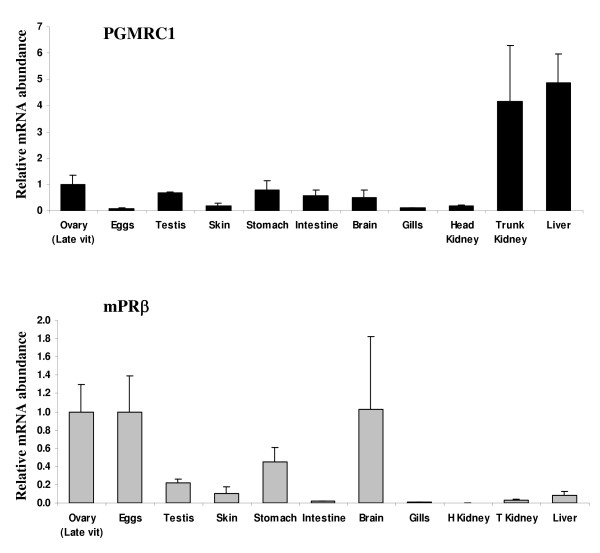
Tissue distribution of PGMRC1 (**A**) and mPRβ (**B**) mRNAs in rainbow trout. Tissues were collected from 3 different females during reproductive period. Ovaries were sampled at late vitellogenic stage. Testes were sampled at stage II of spermatogenesis [31]. The mRNA abundance (mean ± SEM, N = 3 tissue samples originating from different individuals) of each gene was determined by real-time PCR and normalized to the abundance of 18S. For each gene, mRNA abundance was arbitrarily set to 1 for the "ovary" group.

PGMRC1 mRNA was detected in most tissues tested including late vitellogenic ovary (Figure 3). Very low levels were observed in skin and eggs in comparison to other tissues and a strong expression was observed in liver and trunk kidney. MPRβ mRNA was predominantly expressed in reproductive tissues and brain (Figure 3) while low mRNA abundance was observed in most other tissues except stomach (Figure 3).

### Ovarian expression profiles during oogenesis

**Figure 4:**
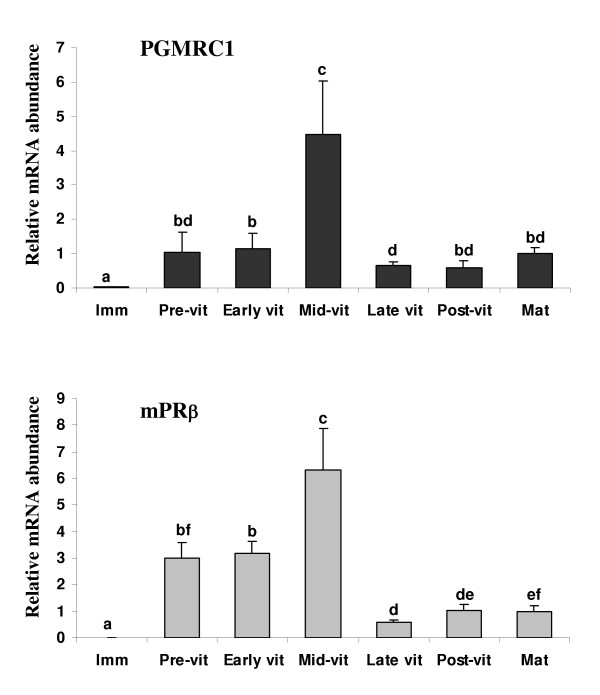
Ovarian expression profiles of PGMRC1 and mPRβ mRNAs during rainbow trout oogenesis (mean ± SEM). Ovaries were sampled from separate females during immature stage (Imm, N = 5 fish, 1000°C. days post-fertilization), pre-vitellogenesis (Pre-vit, N = 6 fish), early vitellogenesis (early vit, N = 6 fish), mid-vitellogenesis (Mid-vit, N = 6 fish), late vitellogenesis (late vit, N = 6 fish), post-vitellogenesis (post-vit, N = 6 fish) and oocyte maturation (Mat, N = 4 fish). The mRNA abundance (mean ± SEM) of each gene was determined by real-time PCR and normalized to the abundance of 18S. For each gene, mRNA abundance was arbitrarily set to 1 for oocyte maturation group. Different letters indicate significant differences between groups (p < 0.05).

**Figure 5:**
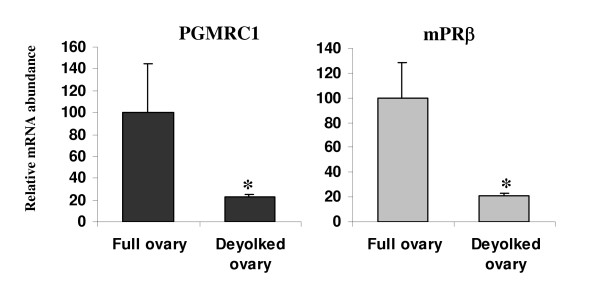
Comparative mRNA abundance of PGMRC1 and mPRβ mRNAs in the rainbow trout intact (N = 3 groups of 25 follicles) or deyolked (N = 3 groups of 25 follicles) post-vitellogenic ovary. The mRNA abundance of each gene (mean ± SEM) was determined by real-time PCR and normalized to the abundance of 18S. For each gene, mRNA abundance was arbitrarily set to 1 in the intact ovary. Asterisks indicate significant differences (p < 0.05).

PGMRC1 and mPRβ mRNAs exhibited similar ovarian expression profiles during rainbow trout oogenesis. Both mRNAs exhibited low levels in differentiating gonads. In contrast, stronger abundance was observed during pre and early vitellogenesis while the highest levels were observed during mid-vitellogenesis. Indeed, PGMRC1 and mPRβ mRNAs were up-regulated during mid-vitellogenesis and down-regulated during late vitellogenesis (Figure 4). During late vitellogenesis, PGMRC1 and mPRβ mRNA abundance was about 15% and 10% of mid-vitellogenic levels respectively. In the preovulatory ovary, PGMRC1 and mPRβ mRNA abundance did not significantly changed from late vitellogenesis to oocyte maturation. In addition, PGMRC1 mRNA abundance was higher than mPRβ mRNA abundance at all ovarian stages tested. Indeed, the number of PCR cycles necessary to observe detectable signal indicated that PGMRC1 mRNA abundance was approximately 5 times stronger than mPRβ mRNA abundance during oocyte maturation. During mid-vitellogenesis, PGMRC1 mRNA abundance was 2 times higher than mPRβ mRNA abundance. Finally, comparative study between intact and deyolked post-vitellogenic follicles showed that both PGMRC1 and mPRβ mRNA abundance was significantly higher in intact follicles than in deyolked follicles (Figure 5).

### Hormonal regulation

**Figure 6:**
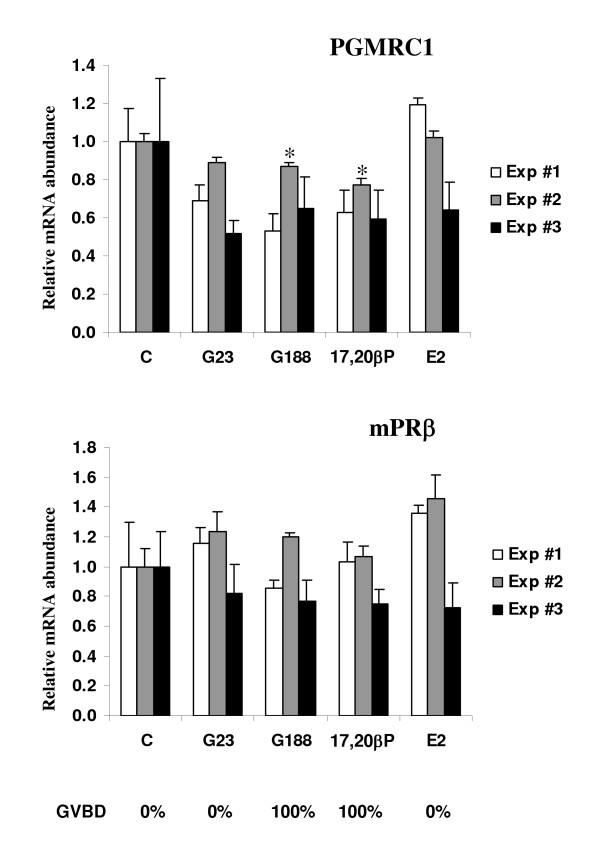
Hormonal regulation of mPRβ and PGMRC1 mRNA abundance in full-grown intact rainbow trout ovarian follicles. The experiment was repeated 3 times and for each experiment, all follicles originated from the same ovary. For each hormonal treatment (**C **= Control; **17,20βP **= 17,20β-dihydroxy-4pregnen-3one, 40 ng/ml; **G23, G188 **= partially purified gonadotropins, 23 or 188 ng/ml; **E2 **= estradiol, 1 μg/ml) the 20 h incubation was performed in triplicates. The mRNA abundance (mean ± SEM, N = 3 groups of 25 follicles) of each gene was determined by real-time PCR and normalized to the abundance of 18 S. For each experiment, the mRNA abundance of each gene was arbitrarily set to 1 for the control group. *: significantly different from the control group of the experiment at p < 0.05. For each hormonal treatment, occurrence of germinal vesicle breakdown (GVBD) observed after 60 h is indicated below the graphs.

After a 60-hour *in vitro *incubation, 100% GVBD was observed in follicles incubated with gonadotropin at the concentration of 188 ng/ml (G188) while the 23 ng/ml (G23) did not induce GVBD. The 17,20βP treatment induced only an incomplete oocyte maturation characterized by remaining traces of germinal vesicle. In contrast, no GVBD nor any visible sign of oocyte maturation could be detected in control or E2 treated groups of follicle. Similar observations were made for the 3 experiments performed. PGMRC1 mRNA abundance appeared lower in G188 and 17,20βP stimulated groups than in control group. However, this difference was significant in only 1 of the 3 experiments performed (Figure 6). In contrast, no significant difference in mPRβ mRNA abundance was observed between control and any hormonal treatment (Figure 6).

### In situ hybridization

**Figure 7:**
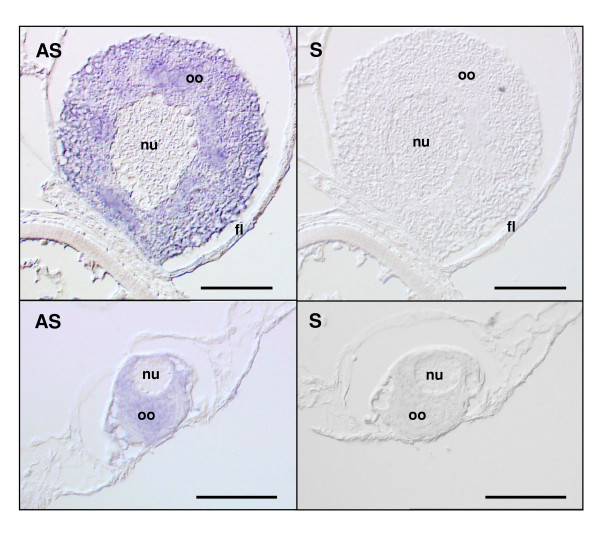
*In situ *hybridization of mid-vitellogenic ovarian tissue in the presence or PGMRC1 antisense (AS) and sense (S) riboprobes. Positive (AS) and background (S) staining of a previtellogenic oocyte are shown. Labels:**n **= nucleus, **oo **= ooplasm, **fl **= follicle somatic layers. Bars represent 50 μm.

Using *in situ *hybridization, the rainbow trout PGMRC1 Antisense probe produced a strong signal in the ooplasm of previtellogenic oocytes that are present in the mid-vitellogenic ovary (Figure 7). No signal was observed elsewhere on ovarian sections. In contrast, the Sense probe did not result in any staining of the oocyte above background levels (Figure 7). Similar observations were made on the 3 ovarian tissue samples originating from 3 different females.

## Discussion

### PGMRC1 characterization and regulation

In the present study, we report the cDNA sequence, ovarian expression profile during reproductive cycle, hormonal regulation and intraovarian localization of PGMRC1 mRNA. PGMRC1 was previously cloned from several mammalian species [24-26,47]. Northern studies showed that the human mRNA was expressed in a wide variety of tissues with a predominant expression in liver and kidney [25]. In other vertebrate species, the cognate cDNA was found in a chicken 2-week old embryo library, a western clawed frog (*Xenopus tropicalis*) whole body library, an African clawed frog embryo library and a zebrafish liver library (Table 1). A PGMRC1 protein sequence was also deduced from the tetraodon (*Tetraodon nigroviridis*) genome (Table 1). PGMRC1 protein (also termed Hpr6 in humans) was also found to be expressed in a panel of human tumor cell lines from breast, cancer and colon and was overexpressed in breast tumors [48]. PGMRC1 protein was also detected in human spermatozoa [27]. However, to our knowledge, no information is available on PGMRC1 mRNA or protein expression in the oocyte of any animal species [49,50], although an inner zone antigen of the rat adrenal (IZA), which is in fact PGMRC1, has been immunodetected in the rat ovary [51-53]. Interestingly, IZA has been also involved in progesterone metabolism, especially 21-hydroxylation [52]. In the present study, we observed PGMRC1 mRNA in many tissues, including ovary. While the strong expression observed in liver and trunk kidney is in good agreement with existing data on PGMRC1 tissue distribution in mammals, the present work is the first report of PGMRC1 mRNA expression in a vertebrate ovary.

In addition, we showed that, within ovarian tissue, PGMRC1 mRNA was localized to the oocyte. PGMRC1 mRNA was detected by *in situ *hybridization in the ooplasm of previtellogenic oocytes (Figure 7) and the comparative expression study performed using intact and deyolked ovarian follicles clearly showed that PGMRC1 mRNA is present in the full-grown oocyte (Figure 5). Indeed, PGMRC1 mRNA abundance was 5 times higher in intact ovarian follicles than in deyolked follicles. Detected levels in deyolked tissue can easily be explained by the presence, within the extrafollicular tissue that remains attached to the follicle, of pre-vitellogenic oocytes that also express PGMRC1 mRNA, as shown by *in situ *hybridization. Surprisingly, no signal was observed by *in situ *hybridization in mid-vitellogenic oocytes. However, during vitellogenesis, oocytes undergo a dramatic volume increase (over 100-fold) that can explain the lack of signal. Indeed, such an increase would necessarily induce a decrease of the signal even if the number of mRNA copies per oocyte remained unchanged.

Interestingly, PGMRC1 mRNA ovarian expression profile during reproductive cycle is similar to mPRβ profile with a maximum during mid-vitellogenesis. Together, our observations suggest that PGMRC1 mRNA is expressed in the rainbow trout oocyte throughout oogenesis and that its abundance in the ovary tends to decrease during late vitellogenesis. Indeed, in the post-vitellogenic follicle, PGMRC1 mRNA abundance is not increased after *in vitro *hormonal stimulation with 17,20βP or gonadotropin. As a matter of fact, we even observed a significant decrease of PGMRC1 mRNA abundance in gonadotropin (188 ng/ml)- and 17,20βP-stimulated full-grown follicles in one experiment. In rat brain, PGMRC1 (also termed 25-Dx) mRNA abundance has been shown to be repressed by progesterone and it was suggested that such mechanism completes an autoregulatory feedback loop, typical of membrane receptors [54]. Even if not significant in all experiments, the observed decrease of PGMRC1 mRNA abundance could correspond to an induction of protein synthesis. Indeed, very low PGMRC1 mRNA levels were observed in metaphase 2 oocytes (unfertilized eggs, Figure 3), suggesting that its has been degraded during oocyte maturation. In mice, it has been shown that some mRNAs that accumulate in the oocyte during oogenesis are degraded during oocyte maturation when the corresponding protein is being synthesized [55].

The bioinformatic analysis of rainbow trout PGMRC1 protein sequence showed the presence of a putative transmembrane domain that was also found in mammalian PGMRC1 protein sequences [24,25]. In addition, PGMRC1 protein was localized to cellular membranes [22,24-26]. In the rainbow trout ovary, PGMRC1 mRNA was only detected in the oocyte. It can therefore be hypothesized that PGMRC1 mRNA encodes for a membrane-associated protein of the rainbow trout oocyte.

Some years ago, a 28 kDa protein, purified from pig liver membranes and exhibiting high binding affinity for progesterone was identified [22]. The protein was partially sequenced and corresponded to the amino acid sequence deduced from the PGMRC1 cDNA that was subsequently cloned from porcine smooth-muscle cells [24]. PGMRC1 cDNA was expressed in Chinese hamster ovary (CHO) cells and led to increased microsomal progesterone binding [29]. In addition, an antibody directed against the recombinant *E. coli *PGMRC1 protein was able to suppress the rapid progesterone-initiated Ca^2++ ^increase in sperm [29]. Besides PGMRC1, a closely related protein termed PGMRC2 was also identified in humans [25] and other vertebrates. All PGMRC proteins exhibit a b5-like ligand-binding domain which is the steroid binding domain [46]. Indeed, these authors have proposed that b5 domains may have served as template for recent evolution of membrane-associated binding domains. According to these authors, PGMRCs may represent an adaptation by which cells could start making use of steroids as triggers for rapid response mechanisms [46]. Similarly to mammalian PGMRCs, the deduced rainbow trout PGMRC1 protein sequence exhibits a clear b5-like domain. It seems therefore likely that rainbow trout PGMRC1, similarly to its cognate mammalian forms, would also exhibit progestin binding capacities. However, this remains to be demonstrated.

### mPRβ characterization and regulation

In the present study, we characterized the cDNA sequence and mRNA ovarian expression profile of rainbow trout mPRβ. The beta form of the recently identified membrane-bound progestin receptor (mPR) class had previously been identified in several species, including human, pig, mouse, African clawed frog, zebrafish [5] and catfish [18]. Tissue distribution studies showed that mPRβ mRNA was predominantly expressed in human brain [5], channel catfish gonads and pituitary [18], and zebrafish gonads, brain and pituitary [18]. In rainbow trout, we observed a predominant expression in reproductive tissues and brain (Figure 3). Our observations are therefore in agreement with existing data in other fish species and the predominant expression in fish ovary strongly suggests an involvement of mPRβ in fish ovarian physiology.

In catfish, mPRβ mRNA levels varied only slightly throughout the cycle and tended to decrease during ovarian growth [56] while they tend to increase during zebrafish follicular development [56]. In the present study, mPRβ mRNA abundance peaked during vitellogenesis. However, significantly lower levels were observed in the preovulatory ovary (late vitellogenic, post-vitellogenic and oocyte maturation stages). In catfish, the mRNA abundance was measured by quantitative PCR in full-grown ovarian follicles hormonally treated for 8 and 16 h with either human chorionic gonadotropin (hCG), 17,20βP or estradiol (E2). Besides a down-regulation observed after a 8-hour incubation with 17,20βP, no significant change in mPRβ mRNA abundance was observed [56]. In zebrafish, similar results were obtained after incubating follicles in vitro with hCG (30 IU/ml) for 5 or 10 h [56]. Using semi-quantitative RT-PCR, a recent study reported a stimulation of mPRβ mRNA levels in zebrafish follicles incubated *in vitro *with hCG [57]. However, it should be noted that this was observed using a very high hCG concentration (100 IU/ml) that induced only 20% oocyte maturation (GVBD occurrence). In the present study, after a 20-hour incubation, no significant difference in mPRβ mRNA abundance was observed between control and gonadotropin or 17,20βP stimulated groups, even though gonadotropin treatment induced 100% GVBD. In zebrafish, a fish species exhibiting the same maturation-inducing steroid than rainbow trout, it was suggested that mPRβ does participate in the control of meiosis resumption [20]. Assuming a similar involvement of mPRβ in rainbow trout oocyte maturation, our observations suggest that luteinizing hormone-induced final oocyte maturation does not require mPRβ mRNA overexpression. It is however possible that a luteinizing hormone induction of mPRβ protein expression occurs. These conclusions are in total agreement with conclusions recently made in catfish and zebrafish [56].

## Conclusion

In the present study, we showed that rainbow trout PGMRC1 and mPRβ mRNAs are expressed in the preovulatory oocyte. Both mRNAs, while belonging to 2 different classes of membrane bound progestin receptors, exhibit similar ovarian expression profiles during the reproductive cycle and are not up-regulated by 17,20βP and maturing gonadotropin. Therefore, the suggested involvement of mPRβ in 17,20βP-induced oocyte maturation in another fish species strongly suggests that PGMRC1 mRNA ovarian expression pattern is compatible with a participation in the control of oocyte maturation. Such participation in the maturation process would probably imply a regulation at post-transcriptional levels (e.g increase of PGMRC1 protein levels).

## Authors' contributions

BM performed sequencing, tissue collection and real-time PCR experiments. TN participated in the real-time PCR study and performed the *in situ *hybridization experiment. AF participated in the writing of the manuscript, the design of the study and its coordination. JB drafted the manuscript and participated in the design and coordination of the study.
